# Advances in high throughput cell culture technologies for therapeutic screening and biological discovery applications

**DOI:** 10.1002/btm2.10627

**Published:** 2023-12-04

**Authors:** Hyeon Ryoo, Hannah Kimmel, Evi Rondo, Gregory H. Underhill

**Affiliations:** ^1^ Bioengineering Department University of Illinois Urbana‐Champaign Urbana Illinois USA

**Keywords:** high‐throughput, microfabrication, organoid, organ‐on‐a‐chip

## Abstract

Cellular phenotypes and functional responses are modulated by the signals present in their microenvironment, including extracellular matrix (ECM) proteins, tissue mechanical properties, soluble signals and nutrients, and cell–cell interactions. To better recapitulate and analyze these complex signals within the framework of more physiologically relevant culture models, high throughput culture platforms can be transformative. High throughput methodologies enable scientists to extract increasingly robust and broad datasets from individual experiments, screen large numbers of conditions for potential hits, better qualify and predict responses for preclinical applications, and reduce reliance on animal studies. High throughput cell culture systems require uniformity, assay miniaturization, specific target identification, and process simplification. In this review, we detail the various techniques that researchers have used to face these challenges and explore cellular responses in a high throughput manner. We highlight several common approaches including two‐dimensional multiwell microplates, microarrays, and microfluidic cell culture systems as well as unencapsulated and encapsulated three‐dimensional high throughput cell culture systems, featuring multiwell microplates, micromolds, microwells, microarrays, granular hydrogels, and cell‐encapsulated microgels. We also discuss current applications of these high throughput technologies, namely stem cell sourcing, drug discovery and predictive toxicology, and personalized medicine, along with emerging opportunities and future impact areas.

**Translational Impact Statement**

This review explores the spectrum of techniques used for high throughput cell culture and their applications in stem cell differentiation, drug discovery & predictive toxicology, and personalized medicine, along with emerging opportunities.

## 1 INTRODUCTION

There is a pressing need for better therapeutic screening and disease models. In order to advance our understanding of the biology of disease models and provide better treatments to patients, the development of improved screening platforms that better recapitulate the complexity of in vivo tissues, within the setting of controllable and reproducible in vitro platforms is essential. High throughput screening (HTS) is a methodology of systematically screening large numbers of conditions for a single, or a defined small set, response, or mechanism to identify conditions of interest, or hits, for further study.^1^ HTS systems favor assay miniaturization, reducing the quantity of reagents and cells required for testing per condition, while increasing the number of conditions tested in a single experiment. The assay formats that work best for HTS are those without wash steps, such as homogeneous or additive tests, in which recent advances in liquid handling, robotics, and improved imaging methodologies can be utilized to their greatest capability to produce reliable and faster results.^2^ The amount of information generated from a HTS experiment can be immense, so process flow and information management of data becomes of utmost importance in order to reproducibly extract meaningful assessments.^3^ Overall, HTS is a fantastic tool for broadly probing large numbers of conditions or compounds in a resource or library, and can serve as a data‐efficient method to identify hits, as well as better qualify and predict responses for preclinical applications in unbiased experiments.

**FIGURE 1:**
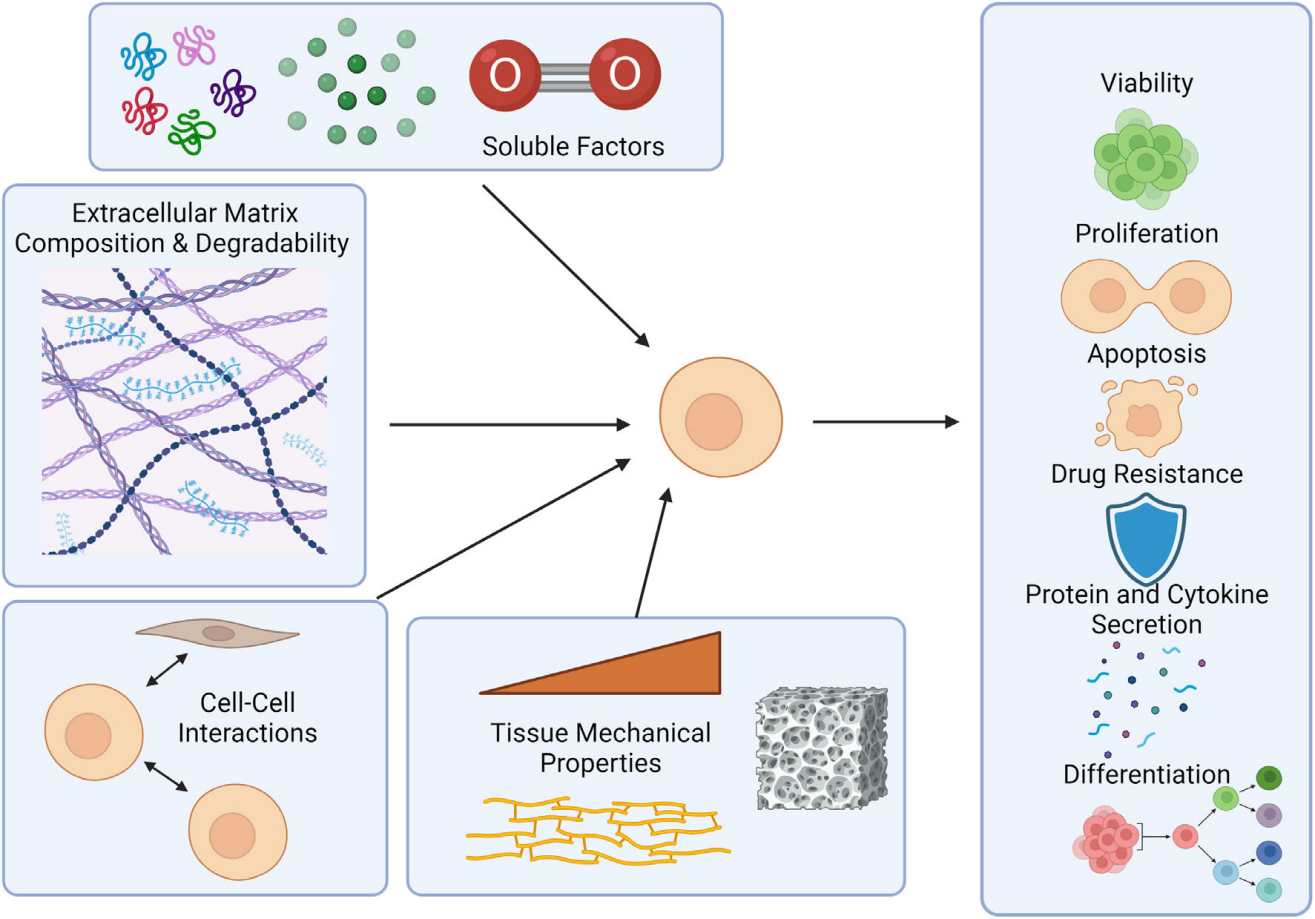
A schematic illustration of the tissue microenvironments' signals to cells, including soluble factors (nutrients and signals), extracellular matrix composition and degradability, interactions with neighboring cells, and tissue mechanical properties (i.e., stiffness, porosity, and elasticity). These signals to cells are then reflected in the cellular responses such as viability, proliferation, apoptosis, drug resistance, protein and cytokine secretion, and differentiation.

High throughput (HT) methodologies can provide researchers with a more complete picture of cellular responses and enable the identification of “hit” compounds that can be subsequently tested in mechanistic experiments together with highlighting potential new avenues of study. Cell‐based in vitro investigations enable the precise control of multifactorial culture conditions, which allows for systematic determination of the specific contributions of each factor. In vivo, the tissue microenvironment provides structural support for the cells and presents biochemical and biophysical cues to instruct the cells' responses and behaviors.^4^ The cells actively interact with their environment sensing tissue stiffness and elasticity,^5^, ^6^, ^7^, ^8^, ^9^ extracellular matrix composition and degradability,^6^, ^7^, ^10^ cell–cell interactions,^11^, ^12^, ^13^ and soluble factors (nutrients and growth factors)^4^, ^14^, ^15^ which can be altered in disease and promote further disease progression^5^, ^16^ (Figure 1). Although cell functional responses are collectively defined by the combination of these cues, simultaneous presentation within a single culture model is often too complex to implement, and can obscure the contributions of each component to the response. HTS systems enable researchers to test many combinations of microenvironmental signals in parallel, as means to deconstruct the influence of changing individual components and the specific effect of increasingly complex combinations. In addition, due to the breadth of conditions that can be assessed within an experimental workflow, HT culture systems can aim to better recapitulate the heterogeneity of the tissue microenvironment in vivo, which is relevant to both intertissue and interpatient variability, an especially helpful feature with the recent legislation allowing in vitro testing to replace animal studies for preclinical qualification of new drugs for FDA approval.

While there are many benefits to be gained from high throughput cell culture systems, there are many challenges that must be overcome. HT systems must be reliable and reproducible, requiring uniformity among conditions, which can represent a challenging optimization requirement in cell culture settings. Additionally, assay miniaturization is important in cell culture screening systems, as reagents and cells can be a limiting resource. Incorporating exogenously delivered microenvironmental cues introduces new challenges to the design of HT cell culture systems, as not only the base cell culture conditions, but also microenvironmental signal delivery, must remain uniform between conditions. Simplifying the creation and handling of these HT systems is an important consideration together with the determination of how to integrate nascent automation technologies for cell culture. Finally, increasing complexity in these HT cell culture systems increases the data available and presents challenges in maintaining throughput and reproducibility throughout the multiple steps of imaging, processing, and data analyses, especially in three‐dimensional systems. In this review, we will cover how the field has addressed these challenges and developed HT cell culture systems capturing different portions of the microenvironment and observing a range of cellular responses to further science. HTS can be divided by the dimensionality in which cells experience their microenvironment (including other cells). Two‐dimensional systems refer to the cells that interact with their environment and each other on one plane, such as typical adherent cell culture conditions. Three‐dimensional HTS incorporates more spatial complexity by enabling cells to experience their environment in all three‐dimensions (*x*,*y*,*z*) and can better replicate the three‐dimensional nature of tissue in vivo. We will first explore two‐dimensional HT cell culture techniques, focusing on multiwell microplates, microarrays, and microfluidics. In the next section, the additional complexity of spatial organization will be explored in three‐dimensional HT cell culture systems. This section will include both unencapsulated and encapsulated cell culture systems, including multiwell microplates, microwells, hanging droplet, micromolds, microarray deposition, micropillar/well chips, granular hydrogels, and cell‐encapsulated microgels. There are some HTS platforms that serve as the foundation for both two‐dimensional and three‐dimensional analysis strategies, we have separated these by their dimensionality, and where appropriate we have separately discussed fabrication and implementation aspects specifically relevant to the distinct strategies. Finally, we discuss current applications for HT cell culture platforms, including directed stem cell differentiation, drug discovery, personalized medicine, and some emerging opportunities in the HT cell culture field.

## 2 TWO‐DIMENSIONAL HIGH THROUGHPUT CELL CULTURE SYSTEMS

There are three main techniques used in two‐dimensional high throughput culture systems: multiwell microplates, microarrays, and microfluidics. All three of these systems enable the exploration of many conditions in one single experiment and typically produce multiplexed readouts that can be utilized to systematically examine biological phenomena. A summary table of these techniques with examples of experimental screenings and responses from literature can be found in Table 1. Each of these methods also has three‐dimensional applications for cell culture and will be revisited in Section 3.

**TABLE 1:** Two‐dimensional high throughput systems including screened variables, the cellular responses measured, and example references for further reading.

Technique	Screened variable	Measured response	References
Multiwell plate	Soluble factors	Cytotoxicity	21, 27
		Cell function	23, 25, 26, 27
Microarrays	ECM components	Proliferation	34, 45
		Differentiation	33, 37, 41, 42, 52, 55
	ECM components and Stiffness	Cell Function	46, 48
		Differentiation	38, 53, 54
		Cytotoxicity	60, 61
	Geometry	Cell Function	36, 44
		Differentiation	56, 57
Microfluidics	Space	Migration	64, 65
	Soluble factors	Cytotoxicity	68, 69, 70

### 2.1 Multiwell microplates

**FIGURE 2:**
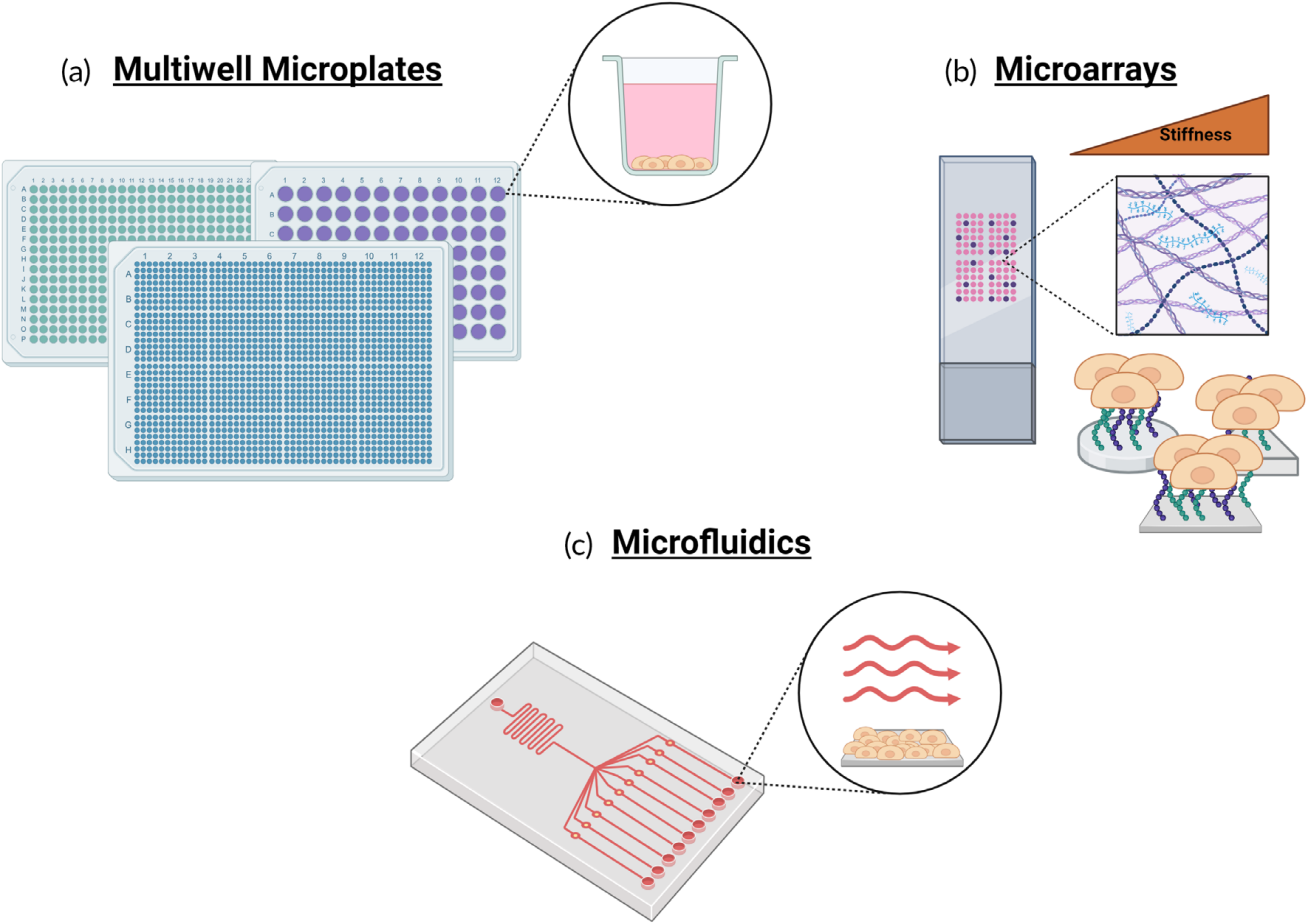
An illustration of the two‐dimensional high throughput cell culture techniques included in this review: (a) multiwell microplates, (b) microarrays, and (c) microfluidics.

Multiwell microplates lend themselves to high throughput (HT) or content (HC) screening applications due to their versatility, efficiency, and ease of use, especially in toxicity and compound screening situations. High‐content screening refers to the combination of HT screening experiments with automated microscopy and quantitative image analysis of multiplexed single‐cell data.^17^ They enable scientists to directly compare many different conditions or compounds on the same plate with low variability. The smaller well sizes allow for a reduction in reagent volumes, increasing the efficiency of experiments and reducing cost per test point. 96‐well and 384‐well plates are most commonly used for HT or high‐content screening as they are supported by most instruments and manufacturers, however, there are 1536‐well plates that are used by labs with more specialized equipment (Figure 2a).^18^ Due to their universality, there are many possible design details to consider when developing a HT assay, including well shape, plate color, bottom shape, and surface treatments.^18^

Many assays have been developed to screen for different markers of interest in a variety of cells in the response to possibly toxic compounds. These assays typically include a measure of cell viability or cytotoxicity as well as a phenotypic or cellular activity marker. Multiwell microplates are very useful in assaying cellular responses to soluble treatments and identifying potential compounds for further study. Multiwell microplates are typically used to study the impact of compounds on cell viability and phenotypic markers or cellular activity. Using fluorescent reporters in situ and post‐culture processing (such as supernatant analysis), researchers can capture information on the impact of the compounds on cellular responses in a HT manner. These readouts can include organelle morphology and function,^19^, ^20^, ^21^ membrane voltage and ion dynamics,^22^, ^23^ and cell‐specific markers including maturation markers,^19^ neurite outgrowth,^23^, ^24^ intracellular triglycerides and lipid accumulation,^25^ and IL‐1B secretion and inflammasome activation.^26^ Grimm et al. established multiple 384‐well toxicology screening platforms to identify toxic compounds to iPSC‐derived cardiomyocytes' and hepatocytes' cellular activity. The platforms measured cell viability, mitochondrial integrity, and reactive oxygen species formation for both cell types, while specifically measuring cardiomyocyte beat frequency and intracellular cAMP levels and hepatocyte lipid accumulation and cytotoxicity in response to compounds.^27^

While most assays are developed using a primary tissue culture treatment and sometimes a secondary cell‐specific adhesive coating,^18^ some systems have been developed to further influence the environment experienced by the cells. Groups have created systems to coat the bottom of the wells with a hydrogel to control the stiffness^28^ and even observe markers such as pH and glucose.^29^ Another avenue of environmental control is level of oxygenation in the wells through PDMS inserts^30^, ^31^ or a microfluidic device.^32^

### 2.2 Microarrays

While multiwell systems are a useful tool for the high‐throughput analysis of soluble cues effects on cells, microarrays offer a way to measure the effects of insoluble microenvironmental cues in a high‐throughput and resource‐efficient manner.^33^ Microarrays are an array of dimensionally uniform spots of single or combinations of microenvironmental cues, typically extracellular matrix (ECM) proteins or growth factors, upon which adherent cells are seeded and cultured (Figure 2b).^33^, ^34^, ^35^ These spots are commonly made through robotic microcontact printing,^33^, ^34^, ^35^ PDMS^36^, ^37^ or silicon^38^ stamping, or specially designed slides.^39^ In order to control cell localization to printed islands, the nonprinted surfaces should not support cell adhesion,^35^ including surfaces such as synthetic hydrogels (i.e., PEG,^38^, ^40^ PDMS,^41^ or polyacrylamide^33^, ^35^, ^37^), glass,^37^, ^42^, ^43^ plastic well plates,^34^, ^44^ or areas of super hydrophobicity.^39^ This creates islands of cells, spatially separated from other conditions, but experiencing the same media and culture conditions allowing for a more direct comparison of microenvironments in a compact and resource‐efficient system. These micropatterned islands can vary in size from singular cell size (~50 μm)^36^ to several hundred cells (1 mm)^39^ and shape including circular^33^, ^34^, ^35^, ^36^, ^37^, ^38^, ^40^, ^42^, ^43^ (most common), square,^36^, ^39^, ^41^ oval,^36^, ^44^ and triangular.^36^ These microarrays have been used to explore a variety of different cell behaviors, including stem cell maintenance and differentiation control/patterning, epithelial to mesenchymal transition (EMT), hypoxia response, drug responses, and disease progression.

Microarrays are extremely useful in investigating the impact of the presented ligands and environmental cues on cellular phenotypic responses. Platforms combining ECM proteins with signaling molecules^34^, ^45^ or stiffness^46^, ^47^, ^48^, ^49^ have been used to study the microenvironment's impact on cellular functional outputs,^46^ proliferation,^34^, ^45^, ^47^ activation,^45^, ^47^, ^48^ phenotype,^47^, ^48^ H3K9 methylation,^34^ and adhesion profiles.^49^ Other microarrays utilize a single adhesive component for the islands and focus on other areas of phenotypic control, such as cellular geometry^36^, ^44^ or co‐cultures.^50^, ^51^ Stem cell differentiation is another area of interest for researchers using microarrays to determine the impact of ECM proteins, alone^33^, ^52^ or in combination with stiffness^38^, ^53^, ^54^ or signaling molecules,^41^, ^42^, ^55^ or cellular^56^, ^57^ and island^58^, ^59^ geometry on fate decision and patterning. Srivastava et al demonstrated that constraining induced pluripotent stem cells to hydrogel island is enough of a trigger for morphogenesis without exogeneous supplements.^37^ Microarrays can also be used to explore the impacts of microenvironments on cells' responses to external stimuli, like drug treatment^60^, ^61^ and hypoxia.^62^ There are other applications that utilize the microarray platform without the addition of proteins, including hydrophilic islands,^39^ drug‐eluting islands,^40^ lentiviral islands,^63^ and DNA oligonucleotide islands.^43^

### 2.3 Microfluidics

Microfluidic devices are an extremely useful tool in the exploration of cell responses in vitro (Figure 2c). They allow for precise control of inputs and reduce the volumes of reagents and cells needed to perform an experiment. Microfluidic devices also enable the addition of shear stress and flow to the model that enables the 2D in vitro models to better replicate the conditions of in vivo. While the initial development and validation can be more involved than the other methods mentioned here, the power of a well‐established microfluidic device provides another level of control for the HT experiments. Currently, microfluidic devices are not considered HT, but they can nonetheless be important platforms for in vitro culture. As discussed in this review, with continual advances in both fabrication and analysis procedures, there are number of new opportunities and proof‐of‐concept demonstrations that could represent the foundation for further integrating microfluidic platforms with HT experimental designs.

Microfluidic devices are useful tools for modifying the physical properties of the cell culture to observe physical phenomena in cell response like the migration of tumor cells.^64^, ^65^ Microfluidics empower researchers to control the exposure and flow of treatments over cell cultures in a way the other 2D methods covered do not. A HT combinatorial screen in a microfluidic device that contained 8100 separate culture chambers was designed to identify key combinations of factors that induce human iPSC‐derived cardiomyocyte proliferation.^66^ An upstream concentration gradient generator and downstream culture chambers were combined to efficiently determine the proper dosage of resveratrol to protect primary mouse chondrocytes.^67^ Combined chemical and mechanical signal gradients on interspersed culture wells were designed to observe the effects of both drug concentration and shear stress on the viability of skin cancer cells and found that high shear stress has a synergistic effect with dosage on cell viability.^68^ A co‐flow focusing, three‐channel microfluidic device was created that could direct path and width of a reagent or drug stream to spatially selective regions and the directed removal of only treated cells for further imaging and analysis.^69^ A crossed laminar flow microfluidic system that could flow many different cell types to attach to printed microarray islands of Collagen I or antibodies that are then exposed to orthogonal streams of drug treatments enabled the HT assessment of drug and cell type interactions in a single experiment.^70^

### 2.4 Potential limitations of 2D and motivation to move to 3D HTS systems

Two‐dimensional HT culture systems offer robust screening of high numbers of soluble and insoluble effectors on cellular behavior and responses. The flat nature of this culture configuration enables high speed and high content imaging and analysis^71^, ^72^ of typically more homogeneous cells^73^ on defined microenvironmental cues,^74^ and can be more easily scalable.^72^, ^75^ HT 2D systems have been invaluable in the efficient screening of large numbers of compounds for the identification of potential drug targets, such as GPCRs and kinases, and increasing the mechanistic understanding of cellular behavior and response. However, such 2D systems exhibit some limitations that hamper their use as physiological screening systems. Cells cultured in 2D can demonstrate abnormal morphology and polarization^76^, ^77^ and are missing the spatial cues found in their tissue microenvironment including the heterogeneous presentation of tissue^75^ and cell‐secreted^74^, ^75^ ECM signals, 3D interactions with other cells of the same or different types,^74^, ^77^ and soluble factor gradients.^73^, ^76^, ^77^ 2D models have been shown to be less physiologically relevant,^76^ with 3D systems showing decreased drug efficacy,^77^, ^78^ differential gene expression,^73^, ^75^, ^77^, ^78^ and differential environmental responses^75^, ^78^ when compared to 2D. The decreased drug efficacy property highlights an important benefit to 3D HT culture systems, as this more physiologically relevant screening system could reduce the false hits that lead to clinical trial failures, saving resources to pursue more effective and safer treatments. Three‐dimensional culture systems enable a more spatially complex presentation of microenvironment to cells; however, there is increased challenge in scalability and high‐content imaging and analysis, detailed in Section 4.3. Most HT imaging and analysis pipelines are well established and developed specifically for 2D HTS applications, so the increased data and complexity of 3D HTS systems requires additional optimization efforts to establish and maintain the throughput and reproducibility of the experimental processes.

## 3 THREE‐DIMENSIONAL HIGH THROUGHPUT CULTURE SYSTEMS

### 3.1 Scaffold‐free spheroid assembly techniques

While two‐dimensional in vitro systems are a great high throughput tool for the analysis of cellular responses, these responses often differ from in vivo findings. Three‐dimensional approaches can help recapitulate in vivo‐like three‐dimensional tissue microenvironments while still providing a HT experimental platform. One of the most common 3D systems employed is simple spheroidal cultures. Cell spheroids are 3D aggregates of cells that can better replicate the spatial signaling and cell–cell interactions of the tissue microenvironment.^79^, ^80^, ^81^ These cell aggregates can be made from a single cell type, a combination of cell types, or composed of self‐organizing stem cells.^79^, ^80^, ^81^, ^82^ The diameter of these spheroids is important, as larger spheroids can develop a necrotic core when above the oxygen diffusion limit, which can be used to replicate the oxygen dynamics and hypoxia of solid tumors.^81^, ^83^, ^84^ When aggregated, the cells interact with each other and their secreted extracellular matrix to better recapitulate in vivo responses, such as drug resistance and organization.^81^, ^83^ There are several ways of creating scaffold‐free spheroids for HT analysis, including hanging droplet, ultra‐low attachment culture plates, and microwells (Table 2).

**TABLE 2:** Three‐dimensional *scaffold*‐*free* high throughput systems including screened variables, the cellular responses measured, and example references for further reading.

Technique	Screened variable	Measured response	References
Hanging droplet	Soluble factors	Cytotoxicity	88, 89, 90, 91, 92
		Cell function	89, 92
	ECM components	Cell function	87
Multiwell plate	Soluble factors	Cytotoxicity	93, 94, 95
		Cell function	94, 95, 96
Microwells	Soluble factors	Cytotoxicity	98, 99, 100, 103
	Spatial confinement	Differentiation	102, 103, 104
Microfluidics	Soluble factors	Cytotoxicity	106, 107, 108, 109

#### 3.1.1 Hanging droplet fabrication

**FIGURE 3:**
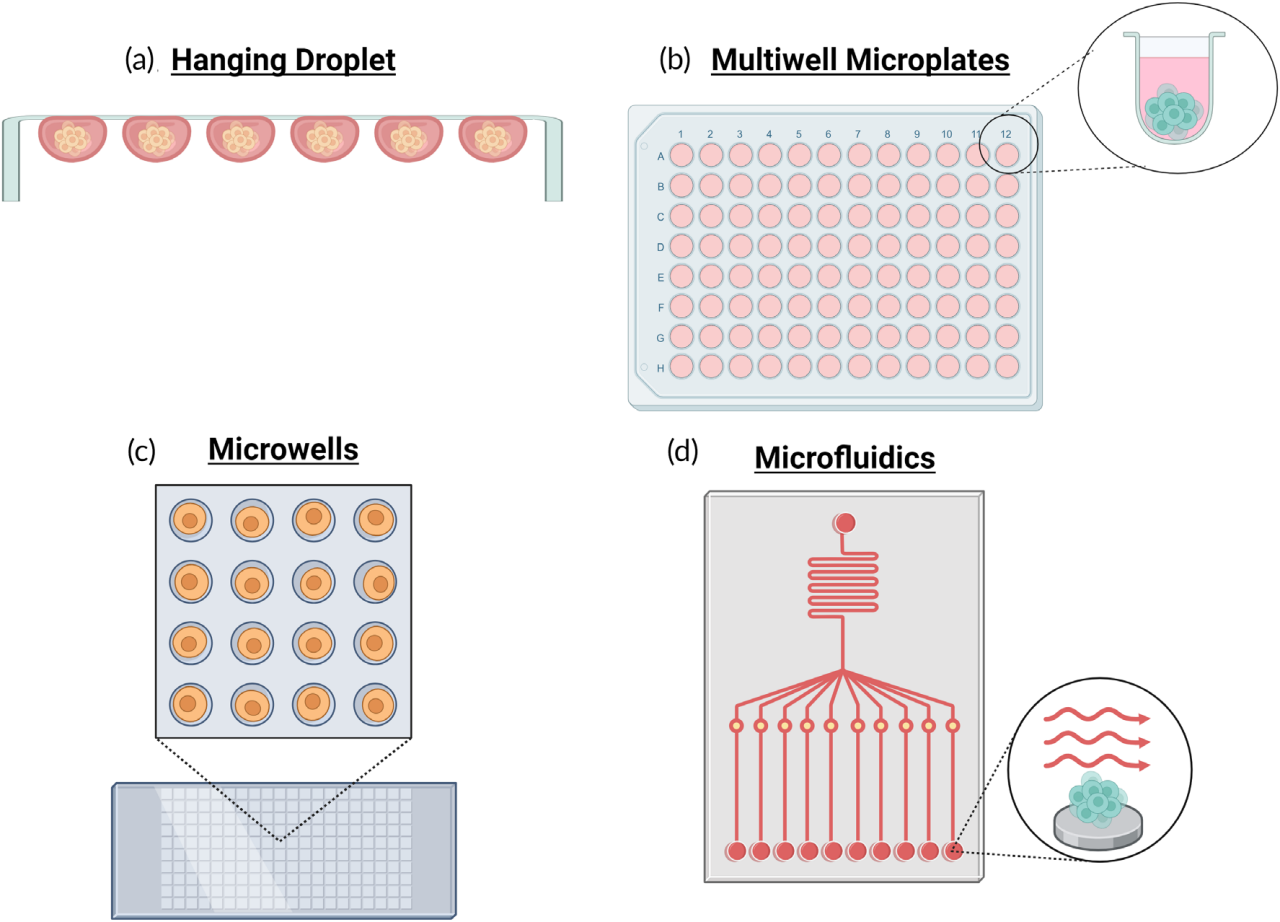
A graphical representation of the scaffold‐free three‐dimensional high throughput cell culture techniques covered in this review includes (a) hanging droplets, (b) multiwell microplates, (c) microwells, and (d) microfluidics.

The hanging droplet method involves small volumes of cell suspension added to a surface and flipped upside down to use gravitational settling to collect the cells at the bottom of the droplet and encourage the cells to adhere to each other and form spheroids (Figure 3a). While the creation of spheroids can be parallelized and sufficiently HT using this technique, the collection and extended culture of the spheroids is difficult, although there are ways of improving the throughput.^81^ Tung et al. created a 384‐well format hanging drop culture plate where spheroids can be cultured long term and treated with drugs using a commercial liquid handler^85^ along with a complementary transfer and imaging plate that worked to capture the spheroids for imaging or easy collection for flow cytometry analysis.^86^ In a different approach, a system similar to 2D microarrays was created for 3D hanging drop culture where cell‐free tissue fragments were combined with cells to explore the impact of different tissue microenvironments on spheroid dynamics and cell behavior in a HT manner.^87^ To address the inherent handling challenges, a digital microfluidic device was designed to enable the controlled culture of hanging drop spheroids with the possibility of media changes and drug treatments without disturbing the spheroids.^88^ Another group utilized a 3D printed platform to create spheroids and dispense them into multiwell plates using direct pipetting for further drug screening, metastasis, and migration analyses.^89^ Platforms with hydrophilic areas surrounded by areas of superhydrophobicity have been used to create spheroids in 100 nL droplets of many cell lines that could then be collected or subjected to drug treatments^90^ and to generate spheroids to be used with an acoustic droplet ejection device to inject a small volume of drug solution into the hanging droplets for screening.^91^ Gheytanchi et al. discovered that the use of spheroids made from two colorectal cancer cell lines showed increased stemness and drug resistance when compared to the 2D monolayer culture.^92^

#### 3.1.2 Ultra‐low attachment culture surfaces

Well plates can be modified to have a surface of hydrogel that blocks cellular attachments and protein adsorption. This forces the cells to form together into an aggregate which can develop into a tighter spheroid over time. The size of the spheroids, like those formed using hanging droplets, is determined by the number of cells in the well. The ultra‐low attachment coatings enable researchers to use the HT potential of multiwell plates in 3D (Figure 3b). 96‐well round bottom ultra‐low attachment plates were used to study the cytotoxicity of FDA‐approved drugs of breast cancer cells alone and in co‐culture with fibroblasts or endothelial cells in 2D monolayers and 3D spheroids and identified many compounds with differential responses.^93^ Ultra‐low attachment 96‐ and 384‐well plates were used to optimize sample handling steps to ensure assay reproducibility and expand the high throughput and content value of the assay through multiparametric characterization of phenotypes which they applied to a library of drug candidates on different cancer types.^94^ A real‐time spheroid tumor growth assay was developed utilizing ultra‐low attachment 384‐wells and constitutively expressed green fluorescent protein to determine the impact of drug treatment on spheroid growth over time with the ability to perform end‐point assays.^95^ Live fluorescent reporters were utilized to visualize real‐time uptake of calcein‐AM (drug efflux pump substrate) and reactive oxidative species creation in developing and treated head and neck cancer spheroids in ultra‐low attachment 96‐well plates, as well as endpoint FACs‐based drug resistance profile of the spheroids.^96^ In a different study, Harimoto et al. used ultra‐low attachment 96‐well plates to create tumor spheroids to be colonized by different bacteria in its necrotic/hypoxic core to observe the impact of bacterial infection on tumor growth kinetics and viability to facilitate development of engineered microbial therapies.^97^

#### 3.1.3 Microwells and microfluidics

Fabricated microwells enable the controlled creation of a greater number of controlled‐size spheroids within a smaller culture footprint (Figure 3c). In this approach, numerous replicate microwells are fabricated inside well plates to increase the number of replicates present in each well. The design of the microwells enables control of the shape, size, and number of cells required to form the spheroids. Microwells also control the location of the spheroids both within the horizontal and vertical planes, making imaging and analysis of the spheroids more tractable. For example, spheroids of a beta cell line were compared to human islet tissue spheroids both created in microwells to control size, and determined that the cell line spheroids acted as a more reliable and consistent assay for antidiabetic drugs while still representing physiological responses observed in the donor samples.^98^ In other work, a 384‐well plate was modified to contain a microwell to confine the spheroid and control its size to form a HT tumor microarray for drug dosage testing on several cancer lines and found that the 3D spheroids better recapitulate the patient responses than 2D.^99^ These results highlight the utility of controlled and uniform spheroid formation and culture to recapitulate in vivo conditions better than 2D platforms and more consistently than patient‐derived models.

Micropatterned 96‐well plates creating nine uniform spheroids per well that could be grown and treated in place, increased the assay efficiency and statistical replicates of each tested compound's effects on the spheroid responses.^100^ PDMS‐stamped microwells in hydrogel formed uniform‐sized multicellular tumor spheroids that could stably be collected and deposited on a 2D surface without losing their geometry, where they would be exposed to drug‐laden graphene oxide‐wrapped gold nanoparticles to identify their spatial dynamics.^101^ Guo et al. found that the use of agarose multi‐well dishes to create spheroids enriched the cancer stem cell population and expression from different cancer cell lines.^102^ A PDMS stamp was used to introduce a micropatterned PEG hydrogel to the bottom of a multiwell plate to generate uniform gastrointestinal organoids that were confined in space for anticancer drug screening and high‐content phenotypic analysis of drug action.^103^ Finally, a PDMS mold to create a PEG hydrogel with microwells containing different sizes of center pillars to further constrain the shape of the spheroid was created to investigate the impact of geometry and stress on early liver development patterning.^104^

Microwells as a platform lend themselves to integration with microfluidic systems as they can be straightforwardly incorporated using overlaid microfluidic channels. Microfluidic systems have been used to isolate spheroids, create spheroids, create a continuous flow culture system, or to deliver gradients of drugs to microwells (Figure 3d). A microfluidic system designed to isolate and entrap individual pancreatic islets into an array with greater spatial resolution enabled the real‐time imaging of their responses to insulin secretagogues or hypoxia.^105^ A system combined microfluidic double emulsion creation of spheroids with microwell culture to provide uniform spheroids and a higher throughput process for in situ imaging and analysis of viability, tumor heterogeneity, and drug response.^106^ In another example, a microfluidic device system was used to seed cells into microwells and determine the quantitative and qualitative viability response of spheroids under limited and continuous perfusion of cytotoxic agents.^107^ Dadgar et al. developed a device with microwells that reduced the number of primary patient‐derived xenograft (PDX) ovarian cancer cells required to create spheroids and test seven drug concentrations and found that the spheroids formed in their microfluidic system had better viability and epithelial cancer phenotype expression than the same microwells in a 96‐well plate.^108^ Finally, a device that establishes a reproducible gradient of drug concentrations across an array of spheroids in microwells without external fluid actuation was created to simultaneously study the effect of drug concentration on spheroid viability and growth.^109^

### 3.2 Scaffold encapsulation techniques

With increasing awareness of the relevance of the cellular microenvironment on cellular behavior, methods and application of 3D cell encapsulation have become a major area of study. Cell viability, proliferation, differentiation, and drug susceptibility have all been shown to be affected by the mechanical and biochemical properties of the culture material, both in 2D and 3D.^8^, ^110^, ^111^, ^112^, ^113^ One of the first studies in biomaterial encapsulation utilized Engelbreth–Holm–Swarm (EHS) mouse sarcoma basement membrane extract, commonly known as Matrigel, to encapsulate healthy and cancerous breast epithelial cells.^114^ Not only was it a highly innovative approach at the time, but this study was also critical in determining the in vivo relevance of 3D culture compared to 2D culture. It showed that normal breast epithelial cells embedded in Matrigel stunted their growth at sizes comparable to the acini in vivo, while 2D cultured cells kept proliferating.^114^ Since then, multiple studies have used Matrigel as an encapsulating material for 3D culture, particularly for organoid development, including lung,^115^, ^116^ liver,^117^ gut,^118^, ^119^ and brain organoids.^120^, ^121^, ^122^ More refined biological materials such as collagen,^123^, ^124^, ^125^ hyaluronic acid (HA),^126^, ^127^, ^128^ and gelatin^129^, ^130^, ^131^ have also been successfully adapted to support 3D cell culture, providing a more controlled and reproducible extracellular microenvironment. Highly defined, bioinert materials such as alginate,^132^, ^133^ dextran,^134^, ^135^, ^136^ and polyethylene glycol (PEG)^137^, ^138^, ^139^, ^140^ can also be used to provide greater control over the degradability of the encapsulating gel and the concentration of cell adhesion sites. As the gelation techniques and the material properties of these encapsulating gels are becoming increasingly well‐established, the translation of these into a HT setting has become a growing field (Table 3).

**TABLE 3:** Three‐dimensional *scaffolded* high throughput systems including screened variables, the cellular responses measured, and example references for further reading.

Technique	Screened variable	Measured response	References
Multiwell plate	Soluble factors	Cytotoxicity	71, 141, 142
		Cell function	143
	ECM components	Differentiation	147, 148, 149
	Porosity	Viability	151
Micromolding	ECM components	Cell function	159
	Mechanical stimulus	Differentiation	164
	Cellular heterogeneity	Proliferation	154
Microcontact printing	Soluble factors	Cytotoxicity	167, 169
		Differentiation	168
	Cell density	Cytotoxicity	170
	ECM components	Differentiation	171
	Mechanical stimulus	Differentiation	172
Micropillar/well chips	Soluble factors	Cytotoxicity	180, 181, 182
		Differentiation	178, 183, 184
	Cell density	Differentiation	178
Microgel encapsulation	Cellular heterogeneity	Differentiation	196, 199
Granular hydrogel scaffold	ECM components	Cell function	220

#### 3.2.1 Multiwell plates

**FIGURE 4:**
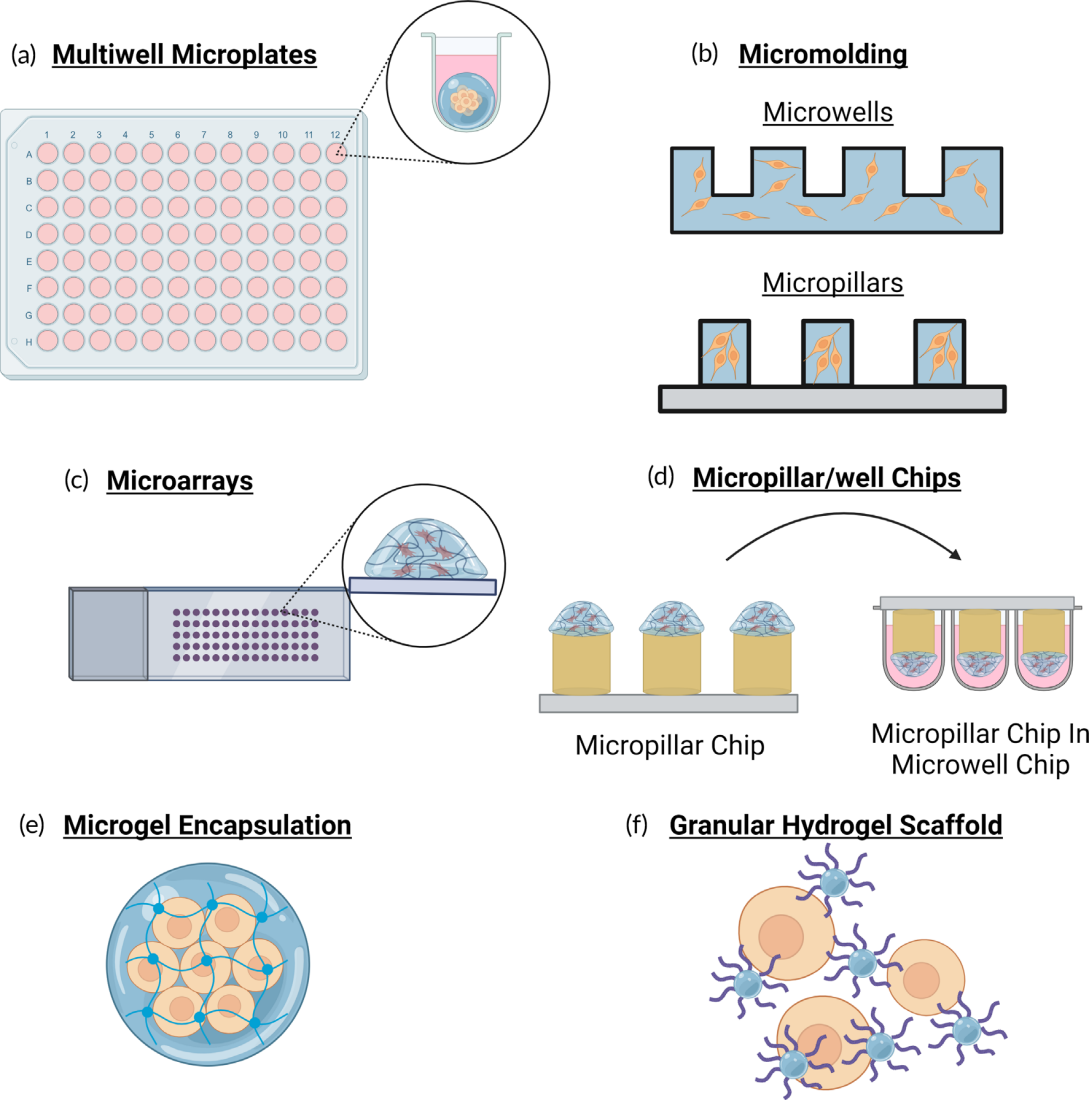
A schematic illustration of three‐dimensional scaffolded high throughput cell culture techniques including (a) multiwell microplates, (b) micromolding, (c) microarrays, (d) micropillar/well chips, (e) microgel encapsulation, and (f) granular hydrogel scaffolds.

The most intuitive technique for HT 3D encapsulated cell culture work is the direct use of multiwell plate or microwells to support the biomaterial and maintain distinct soluble environments for each condition. In this approach, the hydrogel material and cells can be directly added to 96‐, 384‐ or even 1536‐well plates using a liquid handler, with cell culture medium being added on top (Figure 4a). For example, Ramaiahgari et al. employed a liquid handler to distribute HepG2 cells resuspended in Matrigel on 96‐well plates. They observed that the HepG2 cells cultured under these conditions regained lost hepatocyte functions. These liver‐relevant spheroids were exposed to 12 different drugs at multiple concentrations and a higher sensitivity in the identification of hepatotoxic compounds compared to 2D culture was observed.^141^ Malakpour‐Permlid and Oredsson cultured fibroblasts in polycaprolactone fiber meshes in 96‐well plates to observe drug dosage effects on normoxic and hypoxic environments, showing that the 3D environment increased the necessary drug concentration to achieve IC50.^142^ Similarly, liquid handling into 96‐ or 384‐well plates has been used with PEG,^143^, ^144^ gelatin^145^ and beta‐hairpin peptide^146^ hydrogels for the purposes of measuring cell activity or cell viability in a HT process. Deiss et al. have also worked towards creating a modular 96‐well plate where porous paper with 96 bioactive‐spots were stacked on top of each other to create an assembled three‐dimensional structure. After HT culture through different medium conditions, the slabs can be individually extracted, enabling the imaging of lower thickness sections and reducing concerns of z‐stack brightness attenuation.^71^

Ranga et al. employed a liquid handler to distribute varied components and amounts of PEG/cell into 1536‐well plates to interrogate combinatorial effects of stiffness, degradability, cell density, ECM components, cell–cell interaction components, and soluble factors in the colony formation and self‐renewal of mouse embryonic stem cells.^147^ This study of 1024 unique conditions consisted of the combination of degradable peptide‐linked PEG monomers with ECM and cell–cell interaction proteins, cells, and the crosslinking enzyme, FXIIIa, into a solution that was quickly distributed to separate wells to be treated with media containing varied soluble factors. The system was well‐suited for in situ screening of fluorescent markers as well as downstream studies of qPCR and flow cytometry. This HT 3D culture work is especially interesting as it varied the content of the encapsulating hydrogel, in comparison to solely the culture medium, setting the roadmap for the important HT interrogation of the cell microenvironment's mechanical characteristics and its ligand content.^147^ This technology was also used for optimizing iPSC generation, identifying ECM parameters of early neurogenesis, and studying mammary epithelial cell polarization.^148^, ^149^, ^150^ Efforts by Conde‐González and colleagues aimed to analyze the effects of a different hydrogel property, porosity, on cell viability by administering different amounts of DMSO, a porogenic solvent, into a 96‐well plate.^151^

#### 3.2.2 Micromolding

Micromolding consists of the use of a solid mold, typically made of polydimethylsiloxane (PDMS), to form hydrogel microwells or, if inverted, micropillars (Figure 4b). One of the first uses of micromolding for HT cell culture encapsulated NIH‐3T3 cells in a methacrylated HA hydrogel.^152^ A PDMS mold with square and circle indents was filled with cells in HA prepolymer solution, inverted onto a methacrylate functionalized glass surface, and photopolymerized to form an array of solid square/circle hydrogel pillars with embedded cells. Since, micromolded cell encapsulated structures have been manufactured with many different materials, including hyaluronic acid,^153^ collagen,^154^ pullulan,^155^ gelatin,^156^, ^157^, ^158^ alginate,^159^ Matrigel,^160^, ^161^ and interestingly, electrospun dextran fibers.^162^ Micromolding has also been used to create arrays of coculture systems. In one study, 3T3‐L1 stromal cells were encapsulated within a micromolded microwell platform. The microwells could subsequently be filled with breast cancer cells to create a HT coculture system.^163^ The molded wells or pillars can be varied within the same array to create differential stimulus and enable multiparameter studies with the same system. For example, a micromolded micropillar array of alginate with varying pillar diameters allowed for the exertion of variable compression forces through the inflation of a single chamber below the PDMS membrane.^164^

Micromolding has also been used to provide other functions over simply providing the mold for the stabilization of the gelling material. Wang et al. created a PDMS mold that also acted as a microstrainer that could capture the crypts and colonoids formed in a separate bulk 3D culture system, while removing smaller aggregates or single cells. The captured crypts could then be encapsulated with Matrigel in situ. This allowed for the parallel imaging and analysis of highly uniform structures.^165^ Yamaguchi et al. deviced a microwell system where the encapsulation material was photodegradable. This system allowed for the solubilization of the desired microwell upon exposure to 405 nm light. The microwells could be filled with a mixture of different single cells and prepolymer solution and gelled to form a single‐cell array. Upon visualization of the desired reporter cell type within a well, the single well could be exposed to light to release this cell of interest.^166^ These encapsulated microwells or micropillars enable the HT interrogation of co‐culture systems, as different cell types can be introduced to the microwells after their molding. Micromolding adds another degree of complexity in a cell's interaction with the microenvironment and increases the in vivo relevance due to enhanced spatial control.

#### 3.2.3 Microarrays

Microarrays, utilizing microcontact and noncontact deposition, a technique introduced for use in 2D HT studies, have also been adapted for creating HT cell encapsulated 3D systems (Figure 4c). Lee et al. used a microarray spotter to create slides with up to 1080 spots of 20–30 nL, containing collagen or alginate along with MCF7 or HepB3 cells. The slides containing HepB3‐encapsulated alginate were then aligned and stamped with a similar chip containing test compounds encapsulated in alginate, allowing for dose–response studies of different compounds in a single slide.^167^ This technology allows for the printing of two chips with different variables (growth factors, small molecules, shRNA),^168^, ^169^, ^170^ that when are stamped together, can potentially create a double‐variable HT experiment. Microcontact printing has also been used with gelatin‐methacrylate (GelMA), where different ECM proteins were mixed into a GelMA and cell prepolymer solution, contact printed onto a glass slide, and crosslinked with UV light to observe changes in human mesenchymal stem cells' (hMSCs) osteogenic differentiation.^171^

The static and open‐surface capabilities of microarray culture platforms also allowed for the creation of a combinatorial stimulus device capable of presenting combinations of growth factors, ligand density, stiffness, degradability, and most interestingly, mechanical stimulation to the arrayed gel‐encapsulated cells. A combinatorial hydrogel array on a glass slide was placed inverted onto three media chambers of different TGFβ concentrations. This system was also linked to a pressure chamber with pistons of different heights, which exerted differential mechanical stimulation onto the hydrogels upon introduction of nitrogen gas. They observed that the increase in mechanical stimulation led to higher hypertrophic chondrocyte differentiation of hMSCs in high stiffness and high ligand density hydrogels.^172^

In contrast to the arraying of 3D droplets on flat surfaces, it is also possible to array bioactive components onto preformed porous 3D structures to achieve 3D cell culture. For example, Floren et al. used electrospun PEG fibers as a three‐dimensional fibrous matrix onto which combinations of ECM proteins were printed using a microarrayer. The printed proteins were retained in the contact points and enabled the attachment and spreading of MSCs, elucidating several microenvironments that enhance early vascular differentiation.^173^ Modified versions of these PEG fibers with printed combinatorial bioactive components were also used for different applications such as the visualization of endothelium integrity and the quantification of smooth muscle cell activation.^174^, ^175^, ^176^

#### 3.2.4 Micropillar/well chips

Micropillar/well chip technology consists of the formation of gels on top of micropillars, which can then be fitted upside down into microwells with cell media, all steps that can be handled with a multichannel pipette or a liquid handler to enable HT work (Figure 4d).^177^ The technology allows for HT control of two different aspects of 3D culture, as the gel material/cell number can be made variable as well as the cell media content. For example, this technology was used to optimize the oligodendrocyte progenitor cell differentiation parameters of hPSCs, utilizing different cell culture densities and soluble factors.^178^ Other advantages of this system include the use of low amounts of reagents and the possibility of changing all cell culture medium at once instead of well by well, as the whole pillar chip can be moved to a new well chip with medium in one step. The technology has currently been applied for metabolism,^179^ toxicology,^180^, ^181^, ^182^ and differentiation^183^, ^184^ studies in 3D.

#### 3.2.5 Microgel encapsulation

Microgel encapsulation of cells consists of the creation of free‐floating hydrogel particles with sequestered cells, making it a highly dynamic and adaptable culture system (Figure 4e). Microgels are increasingly considered advantageous due to their potential use as injectable material to transplant cells.^185^ Microgel encapsulation of cells has surprisingly early beginnings. A 1966 study detailed the encapsulation of lichen cells in polyacrylamide granules to be used as part of a column assay.^186^ In 1992, Hubbell et al. detailed the encapsulation of porcine islets in bioinert and noncytotoxic PEG diacrylate by first incubating the cells in Eosin Y, a photosensitive molecule commonly used for H&E staining, resuspending the islets in PEG diacrylate and triethanolamine and exposing with a 514 nm laser. Since the photosensitive component diffuses out of the islets, the PEG diacrylate is only able to be polymerized in proximity to the islets, preventing bulk polymerization and creating microgels.^187^, ^188^

Microgels can also be made by direct photolithography by placing a photomask on top of a photopolymerizable solution and exposing the solution to the appropriate light source, commonly UV. For example, Koh et al. flowed PEG‐diacrylate and a photoinitiator solution within a microchamber, covered the channels with a photomask, and exposed the platform to UV light, creating rectangular microgels with encapsulated 3T3 fibroblasts, macrophages, and hepatocytes.^189^, ^190^ Panda et al. coupled the photolithography technique with microfluidics to create a process dubbed stop‐flow lithography. With a constant flow of cells in PEG‐diacrylate, they exposed the photomasked microfluidic channel to pulses of UV light to create microgels of rectangular, triangular, and circular shape.^191^ They later demonstrated the possibility of assembling these different‐shaped blocks to form mesoscale, heterogeneous complexes.^192^

Another method of producing microgels is by employing micromolds as a template for the prepolymer solution and cells. Once this prepolymer solution is crosslinked, the microgels can be released from the molds.^193^, ^194^, ^195^ The HT nature of these micromolded microgel systems were put to the test in a study by Chen et al., where a population of microgels containing embryonic stem cells were bulk cultured to differentiate and enriched for the desired phenotype using fluorescence‐activated sorting (FACS). They also explored the possibility of labeling each microgel during the manufacturing and culture process to keep track of the initial culture conditions of the microgels throughout the analysis steps.^196^

The microfluidic formation of microgels can greatly increase the output of microgel formation. The technique consists of the creation of aqueous, prepolymer droplets separated by oil or other immiscible fluids in a uniform and defined manner within microfluidic chips.^197^, ^198^ The high uniformity of the microgels created through microfluidics also increases the uniformity of the amount of cells that get encapsulated within the gel, enabling clonal or single‐cell studies in HT.^199^, ^200^ This uniformity also allowed Chan et al. to produce spheroids of uniform sizes by employing a double emulsion microfluidic system, where the gelation of the inner‐most liquid could be delayed until the single cells formed an aggregate.^201^ Tan et al. showed that a microfluidic, microgel fabrication system could be coupled with a microgel‐trapping device to provide a fixed microarray system that can be later selectively retrieved depending on desired expression.^202^, ^203^ While microgels made with microfluidics can be highly uniform, they require a high level of expertise for use, and thus simpler dropwise methods could be of interest.^204^, ^205^, ^206^

#### 3.2.6 Granular hydrogel scaffolds

Microgels as a scaffold for cell culture is a growing area of study that has recently gained sufficient interest in the biomaterials and tissue engineering fields. Often also called granular hydrogel scaffolds, microscale hydrogel scaffolds are functionalized with bioadhesive ligands to provide a microporous scaffold for 3D cell culture upon assembly (Figure 4f).^207^ The most common methods of fabricating microgels for suitability as a cell culture scaffold are mechanical fragmentation,^208^, ^209^ batch emulsion,^210^, ^211^ microfluidics^212^, ^213^ and photolithography.^214^, ^215^ In contrast to the cell‐encapsulated microgels mentioned previously, these microgels can be made in harsher manufacturing environments as cell viability during polymerization or formation of droplets is not a design consideration, due to the timing of cell introduction which occurs subsequent to microgel fabrication. This allows for a broader range of photopolymerization or temperature‐dependent polymerization conditions, if required. With such fabrication methods, the microgels can exhibit a range of geometries beyond simply spherical, with microgels in the shape of rods,^216^, ^217^ fibers^162^, ^208^ and irregularly shaped^209^, ^218^ microgels all used previously as scaffold material.

Other potential benefits of microgel platforms as the foundation for HT systems comes from their ability to form heterogeneous microenvironments as well as their enhanced capability for manipulation as a suspension (i.e., injection, pipetting). De Rutte et al. created RGD‐linked PEG microgels of variable stiffnesses and intermixed them at different ratios for the culture of human dermal fibroblasts. They observed that the fibroblasts preferentially adhered to the stiffer microgels in these intermixed scaffolds.^219^ While this first demonstration was not high throughput in design, it is conceivable to consider that through the use of scalable culture manipulation strategies such as liquid handling, distinct microgel compositions and ratios could be combined within a HT culture condition platform. Recently, we demonstrated the implementation of such a strategy towards the creation and analysis of 92 heterogeneous microenvironments for the culture of hepatic stellate cells in the context of nonalcoholic steatohepatitis, a condition that is characterized by heterogeneity of cellular microenvironments.^220^

### 3.3 Analytical methods for HT 3D cultures

HT experiments in 3D are currently more limited than their 2D counterparts in the types of biological outputs or analytical methods that can be performed without resorting to time‐consuming processes, which could place a barrier on the throughput scale‐up. While automated image analysis software is well‐developed and available for 2D systems, imaging and analyzing 3D images is much more complex and not as developed. The amount of information collected in 3D systems utilizing optical sectioning is immense and requires automated analysis programs for segmenting and extracting features to be viable for high throughput experiments.^72^ In the case of HT 3D experiments in standardized well plates, the simplest and most reliable method for obtaining quantifiable data is to use a plate reader with fluorescence or absorbance readouts. This makes viability assays such as MTT or CellTiter‐Glo® very popular,^142^, ^144^ with ongoing progress being made in finding substrates that respond to other types of stimuli, such as MMP activity.^143^ Plate imagers are also capable of obtaining z‐stack images of well content, but the imaging and analysis of such data can be time‐consuming if an analytical pipeline is not well‐established. In some cases, these images are simplified by performing an orthogonal projection to obtain the value of interest^147^ or by obtaining a simpler readout such as the 3D cross‐section.^221^

A recent exciting study used a high‐content screening microscope to obtain 10^7^ images from a 2789 compound screen of intestinal organoids in 384 well plates, that were maximum intensity‐projected and quantified to cluster the organoids into seven phenotypes, each with a unique set of genes that were affected by the compound added. Hierarchical interaction scores were used to determine upstream regulators of each phenotype, leading to the identification and in‐depth study of the RXRA gene as highly influential in intestinal organoids' regenerative state.^222^ Other efforts made use of inverted, gold‐coated pyramidal wells to capture cells and allow them to form spheroids within these chambers. These chambers reflected light from an inverted microscope allowing it to perform a specialized version of light sheet microscopy called single‐objective selective‐plane illumination microscopy. The microscope was programmed to automatically identify organoids and perform rapid setup in a single acquisition workflow.^223^ These studies all employed in‐house developed or expensive automated acquisition and image analysis tools, indicating the current necessity of such specialized tools for HT imaging studies of 3D structures. Until such tools become commercially available with straightforward‐to‐use interfaces, it will be difficult to achieve widespread adoption.

For immunofluorescence‐based experimental measurements, 3D culture configurations can be problematic due to the attenuation of fluorescence signal as the *z* distance is increased as well as decreased penetration of staining materials such as antibodies. Heub et al. have created a method of analyzing multiple 3D samples in one slide by creating a compact, paraffin‐embeddable mold with microwells that can be filled with spheroids using a liquid handler. The mold can then be cryo‐sectioned and the sections stained and mounted as a whole rather than individually, enabling the imaging and staining of thin sections over a whole spheroid.^224^ Current developments in tissue clearing methods^225^ will also aid in the adaptation of HT 3D experimentation. Recent work by Blatchley et al. has shown the possibility of uniformly expanding a 3D construct of interest by permeating it with a hydrogel that swells upon light exposure. The expansion decreases signal attenuation while also allowing for sub‐micrometer‐scale resolution imaging.^226^

Interestingly, Leggett et al. have developed a higher throughput method of analyzing 3D traction force in silk‐collagen encapsulated spheroids by using automated imaging methods, establishing image segmentation and filtering routines, and employing a rapid 3D deformation quantification software.^227^ For the growth of HT systems, expanding on the toolkit for the quantification of different cell behaviors such as mechanobiology, as presented here, will be indispensable.

Microarray systems can use the controlled spatial positioning inherent to the array platform, specifically, that the culture droplet is retained unique position, to facilitate the use of nonconventional analytical techniques such as electrochemical readouts of cell viability. In a study by Jeong and colleagues, the gel/cell was placed on top of a gold electrode, connected to another gold electrode, and a reference silver electrode. They observed that as the number of live cells in the gel solution increased, the current between the electrodes did too, enabling the use of this technology for the live measurement of cell viability and hence the cytotoxicity of drugs.^228^, ^229^ In the case of microgel encapsulated cells, it is also possible to perform fluorescence‐activated cell sorting as an analytical method, as each microgel is its own analytical subunit that can be formed to be smaller than the usual size limit of flow cytometry machines.^199^ Additionally, spatial omics is an emerging suite of technologies that could increase the ability to analyze the 3D microenvironment with spatial resolution, highlighting the heterogeneity, evolution, and cellular profiles using bar‐coded RNA, DNA, or fluorescently tagged antibodies to visualize and sequence.^230^, ^231^, ^232^ Spatial omics greatly increases the data generated and incorporates precise spatial resolution. As the amount of data increases with higher throughput systems, methods to analyze the quantified data become increasingly important. Processing the images into usable data is a challenging step for 3D HT system analysis. The amount of information per image, as well as the size of the file, increases significantly in 3D. Most image processing and cell segmentation pipelines commercially available are set up for 2D systems only, with 3D systems much harder to process due to the multiple optical slices obtained during imaging. While raw data is highly valuable, if it is not easily visualizable, key findings could be lost in the noise. The first statistical test that is often performed to observe the quality of the high throughput assay is to obtain the Z‐factor, which compares the standard deviations and means of the negative and positive controls. A Z‐factor value between 0.5 and 1 indicates an “excellent assay”.^233^ On the data analysis side, one of the main ways in which high throughput data can be analyzed is through principal component analysis (PCA). PCA transforms the data into fewer dimensions, summarizing the output variables in the study, called principal components. PCA allows the portrayal of multi‐dimensional data in two dimensions of maximum variability, which in combination with hierarchical clustering, can show phenotypic (in biological contexts) clusters in one easily visualized chart.^234^

## 4 APPLICATIONS AND OPPORTUNITIES

**FIGURE 5:**
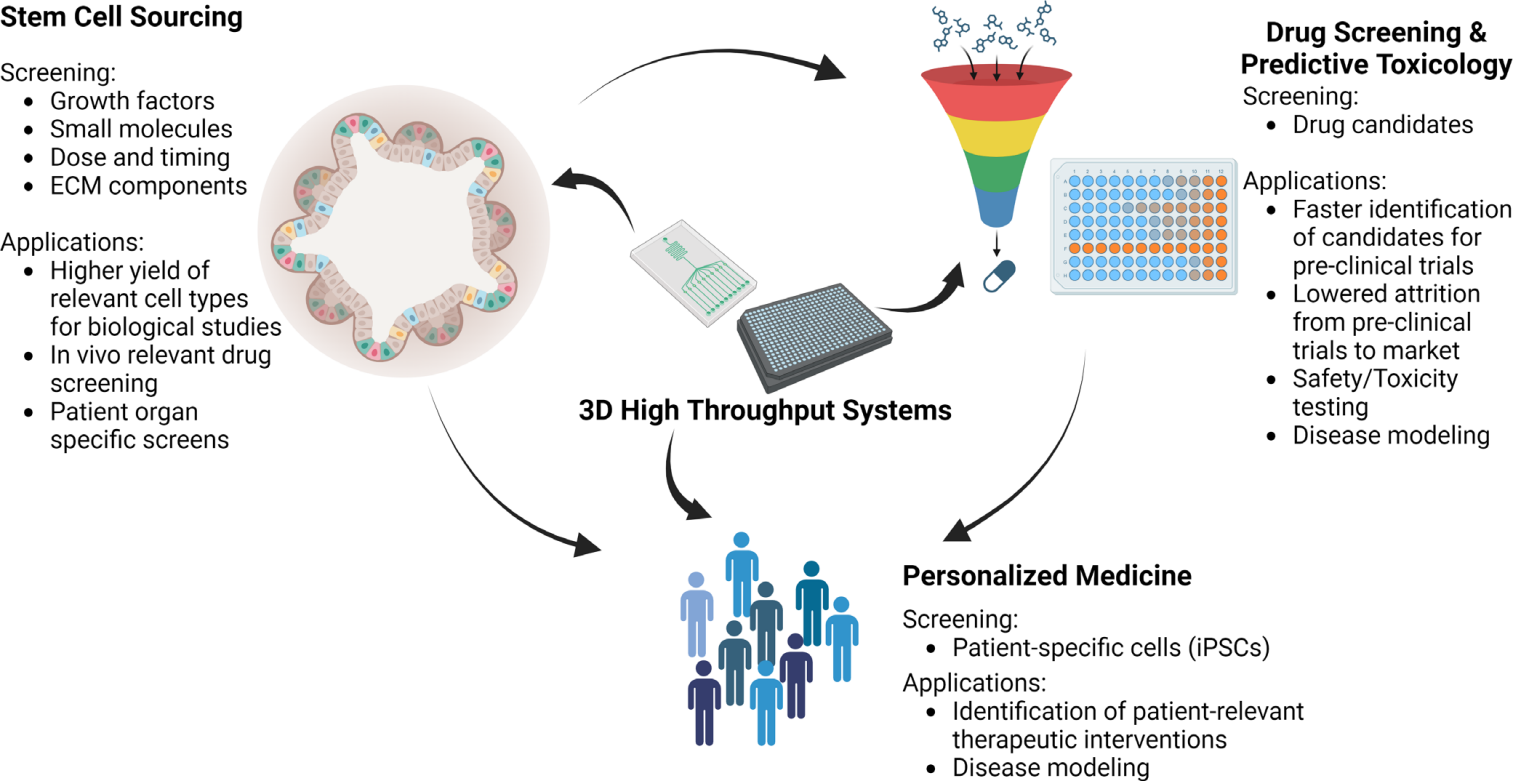
An illustration of past and current translational applications of 2D and 3D high throughput screening systems including stem cell sourcing, drug screening and predictive toxicology, and personalized medicine.

HTS has been pivotal in the empirical testing of various input variables to assess their effect on cellular behavior. In this section, we will explore the past and current translational applications of 2D and 3D cell culture HTS as well as the possible future opportunities within HTS (Figure 5).

### 4.1 Stem cell sourcing

Organoids are defined as 3D structures grown from stem cells that consist of organ‐specific cell types and are able to self‐organize.^235^ Organoids are popular for their potential ability to model development processes, to serve as a drug screening and predictive toxicology system (especially with the recent push to move away from animal testing in both Europe and the United States)^236^, ^237^ and the possibility of becoming transplantable material.^238^ Thus, there is currently an active endeavor to produce reliable and consistent organoids at large scales. Efforts aimed at optimizing organoid systems build upon foundational studies examining the effects of microenvironmental signals on stem cell differentiation and the integration with microtechnology approaches such as microarrays and microfluidics discussed in this review. As an early study in 2D, Flaim et al. performed high throughput mouse embryonic stem cell to early hepatic fate differentiation screening by creating microarrays of combinatorial ECM proteins, identifying the inclusion of collagen I and fibronectin within ECM combinations as an important factor on this differentiation procedure.^33^ A different study in 2D employed a factorial screening microfluidic system where each column was supplied a different ratio of extrinsic factors and each row allowed for the carry‐over of conditioned medium from the previous row. The results obtained from the microfluidic device enabled the improvement of directed differentiation in static cultures.^239^

Scaffold‐free and scaffolded 3D HTS experiments for the improvement of directed differentiation for stem cell sourcing have also been performed. A micropillar–microwell platform study involved the encapsulation of embryonic or induced pluripotent stem cells in Matrigel on top of micropillars that were then inverted onto microwells containing varying concentrations of external factors that influence dendrocyte progenitor or midbrain dopaminergic neuronal cell differentiation. The unique structure of the high throughput system allowed them to regulate dosage, duration, dynamics, and combinations of these factors per individual micropillar, ending up in 1200 unique differentiation timelines analyzed.^178^ Another HT study exploited liquid handling to produce kidney organoids in a fully‐automated manner on 384‐well plates. The high throughput system enabled the dose–response study of CHIR99021, B27, and VEGF on kidney organoid induction and formation from pluripotent stem cells.^240^

HT differentiation screening may serve as a means to evaluate and account for patient‐specific variability in their iPSC differentiation procedures that has been observed.^241^ Further, insights gained from HT organoid differentiation assessment studies will help to inform the optimization of organoid differentiation protocols towards enhanced consistency and improved differentiation outputs. Such advancements will enhance the yield of stem cell sourcing for cell‐based therapeutics in regenerative medicine, and accelerate the adoption of organoid drug screening platforms, while also serving as reproducible models to study development and disease progression.

Another opportunity in differentiation optimization lies in the production of cell‐based or cultured meats. Concerns of animal welfare, land usage, and greenhouse gas emissions are some of the key reasons for the soaring interest in alternative sources of protein.^242^ The production of cell‐based meats involves the differentiation of muscle‐derived cells into mature muscle tissue as well as adipose tissue.^243^, ^244^ HTS of such differentiation procedures could lead to further optimization of the directed differentiation, while aiding in the scale‐up in order to meet market demands and lower cost of production. The ability to source and reliably differentiate stem cells in a more HT manner increases the availability of stem cell models for drug screening, predictive toxicology, and personalized medicine applications.

### 4.2 Drug screening and predictive toxicology

Pharmaceutical endeavors commonly employ HTS to identify relevant compounds within a library that can span millions of distinct compounds.^245^ For one drug to progress to the commercial market stage, thousands of different compounds are initially passed through the drug discovery phase, from which only 250 compounds proceed to the preclinical phase for investigating their mechanism of action, and only five of these ultimately progress to clinical trials.^246^ Across all stages including up to the clinical trial stage, a HTS approach could reduce monetary and time costs. HTS strategies have been instrumental in the development and testing of FDA‐approved drugs for cancer and HIV among other conditions, currently available in the pharmaceutical market.^247^ Furthermore, as mentioned previously, increasing regulation on animal testing and the appreciation for human‐specific disease phenotypes and drug responses, have made the development of medically relevant in vitro models for drug screening imperative. Such developments would not only minimize the requirement for animal models, but could additionally reduce drug screening costs, while further providing human‐relevant data.^248^ HT systems could facilitate the rigorous testing of relevant compounds during the preclinical phases, reducing the number of compounds that do not make it through the clinical trials and thus further reducing costs. A 2009 study from Novartis showed that the number of employees working on HTS projects increased ~50% within a period of 1998–2007, the number of compounds tested had grown more than 10‐fold, with the cost per compound being reduced by approximately 10‐fold within this time period.^245^

The passing of the FDA Modernization Act 2.0 into law authorized sponsors of novel drugs to use alternative techniques to animal testing for safety and effectiveness studies, including in vitro, in silico, or in chemico methods such as cell‐based assays, microphysiological systems or bioprinted or computer models.^237^, ^249^ This law provides a greater potential for these HTS systems to impact drug development, however, increased standardization and validation with gold standard agents is required to maximize their impact and utility. HT screening systems, in particular 3D systems, also provide the ability to predict possible toxicological effects of drugs before they reach clinical trials. A recent study showcased a potential application for this HT toxicity screen by using human iPSC‐derived cardiomyocytes and neurons of different human leukocyte antigen (HLA) donors to create a better representation of the population's genetic makeup to identify potential cardiotoxic and neurotoxic chemicals.^250^ The development of HTS that can identify potential toxicities and adverse effects of compounds early in testing can help avoid failed clinical trials and help focus development on compounds with increased likelihood of clinical efficacy.

As an effort to obtain homogeneous organoids, Renner et al. employed robotic liquid handling techniques for establishment and analysis of midbrain organoids from small molecule neural precursor cells, and demonstrated that this approach facilitated consistent end‐point results. This study demonstrated, using RNA‐sequencing, that there were minimal batch‐to‐batch differences between the organoids formed. Such uniformity allowed for the reproducible evaluation of drug effects, enabling the analysis of dopaminergic neuron‐specific toxicity with minimal variability.^251^ A study by Brandenberg et al. also demonstrated a scalable organoid‐culture technology in which they were able to obtain much more homogeneous gastrointestinal organoids compared to regular Matrigel culture. Due to the microcavity array format of each well, they were able to obtain multiple‐replicate drug screening data from one single well of a 24‐well plate. As expected, the organoids were not as good as single cell‐type derived spheroids in the *Z* score or correlation factor, but these values were still in the acceptable range. They also showed that the organoids exhibited differential drug sensitivities compared to spheroids formulated from established cell lines.^103^ Overall, such efforts incorporating more tightly controlled organoid culture conditions, such as cell numbers and multicellular geometry, can enable reduced output variability and enhance the accuracy of the high throughput assays. These advances paired with improvements in differentiation protocols will take us a step closer to the adoption of in vitro organoid culture as a replacement for animal models and ultimately the reduction of cost and time between the development and release of new life‐saving therapeutics.

### 4.3 Personalized medicine

Personalized medicine refers to the determination of a therapy regimen based on an individual's unique clinical, genetic, genomic, and environmental setting.^252^ With the advent of induced pluripotent stem cells or iPSCs, testing for therapies directly on the patients' cells in organ‐specific contexts ex vivo became a possibility. It is well known that differences in biological factors such as sex, age, and race as well as interindividual variability of conditions such as cancer can lead to clinical differences in treatment outcomes.^253^ As such, a personalized medical intervention could drastically improve patient outcomes. Patient‐derived iPSCs are formed by harvesting common cells such as dermal fibroblasts from a patient and having them express reprogramming factors such as OCT4 and SOX2 through integrative or nonintegrative protein‐encoding strategies in vitro.^254^ The differentiation of these iPSCs into cells of the disease‐relevant context allows for the testing of drugs in a more personalized manner. For example, a study comparing phenotypic and genotypic profiles of gastroesophageal cancer patients' tumors and tumor‐derived organoids demonstrated that the two models exhibited a high degree of similarity. Also, anticancer therapies in ex vivo organoids could recapitulate the patient responses in the clinic, confirming the usefulness of these patient‐derived organoid models.^255^

Kim et al. established 80 unique lung cancer organoids from different lung cancer samples, as well as five different normal bronchial organoids for the creation of a biobank. They observed that these organoids maintained the histological and genetic characteristics of their parent tissues. To test if the organoids could in fact serve to predict patient‐specific drug responses, they performed high throughput drug screening on a subset of the organoids, revealing similar trends of drug sensitivity between these and xenografts derived from the same tissue.^256^ Similarly, Sachs et al. established more than 100 breast cancer organoids from patient samples to create a breast cancer organoid biobank. They also demonstrated that these organoids retained the morphology and phenotype of their original samples. Also similarly, in vitro high throughput drug screening results matched the drug sensitivity observed in xenografts as well as real patient responses.^257^ Such studies have also been performed in micropillar/microwell setups using glioblastoma patient‐derived cells.^258^

In the context of iPSC‐derived cell drug screening, Imamura et al. performed a drug screen on motor neurons differentiated from an amyotrophic lateral sclerosis patient's iPSCs. The patient's condition was caused by a mutation in superoxide dismutase 1 (SOD1). Through the screen, they identified bosutinib as a potential treatment for this subtype of ALS, as it showed increased survival of these differentiated cells. When tested on mouse models of ALS with an SOD1 mutation, bosutinib was also able to modestly extend the survival of the mice.^259^ A great review by Elitt et al. contains a comprehensive list of similar screening tests performed on iPSCs.^260^

### 4.4 Emerging opportunities

Advancements in biomaterial production and capabilities show promising translational opportunities toward the high throughput study of cell behavior. One of these advancements is the 4D control of the extracellular matrix. Cells are dynamic machines that can change their phenotype and outputs based on the various mechanical or biochemical inputs provided to them in their current context.^261^ Thus, temporal control of the cellular microenvironment could expand our understanding of these dynamic processes, elucidating the more nuanced aspects of differentiation, immune response, angiogenesis, matrix deposition, and so forth.

Khetan et al. demonstrated the feasibility of patterning hydrogels in situ during 3D cell culture. They first encapsulated human mesenchymal stem cells by crosslinking the hydrogel by spontaneous reaction of acrylated hyaluronic acid with dithiol crosslinkers, creating a relatively soft gel. After establishing the culture, they stiffened the hydrogel only on one side by photopolymerization, enabling the bonding of the remnant acrylate groups.^262^ Conversely, Kloxin et al. demonstrated that hydrogels could be softened and bio‐inactivated with temporal control by polymerizing the hydrogel using spontaneous reactions and adding photo‐cleavable crosslinkers and cell‐adhesive ligands. Upon exposure to 365 nm light, the photo‐cleavable crosslinkers or cell‐adhesive ligands would dissociate, softening the gel or inactivating the hydrogel. By using confocal laser scanning microscopes, they were able to create and perform these procedures in a spatially controlled manner within the hydrogel.^263^

Similar technologies have now been applied to higher throughput contexts. Mabry et al. cultured dynamically controllable hydrogels in 96‐well plates and modified the hydrogels' RGDS concentration and stiffness in a time‐controlled manner, allowing them to quantify valvular interstitial cells' aspect ratio and activation phenotype for 15 different dynamic hydrogel conditions.^264^ More recently they also showed the possibility of guiding intestinal organoid crypt formation by softening specific areas around the encapsulated organoid.^265^ Batalov et al. also demonstrated the highly spatially controlled addition of bioactive growth factors by using UV light and photolabile groups that expose an oxime ligation motif.^266^ Rapp and DeForest have also created photodegradable materials that cleave upon exposure to three different wavelengths. Until now, most photodegradable chemistries relied on near UV light, but this study expanded the demonstrated capabilities to include green and red wavelength light.^267^

There is also a growing body of literature that has explored the utility of different stimuli‐responsive functionalities. These can range from magnetic substrates,^268^ pH sensitivity, ion presence, enzymatic activity to heat, and many more.^269^ Interestingly, Badeau et al. created 17 different cross‐linker architectures that degraded upon YES/OR/AND combinations of three distinct stimuli: reducing conditions, matrix remodeling proteins, and UV light. This presented an exciting opportunity for enhancing the control of the time and space in which biomaterials exhibit responses such as softening or protein or drug release.^270^, ^271^ We believe that the high throughput coupling of these spatially defined light exposure technologies as well as other stimuli could enable exciting high throughput experimental studies of cellular dynamics that are not only stiffness‐dependent but also bioactivity.

Organ‐on‐a‐chip (OOC) systems are also an emerging opportunity for HT screening and development. OOC systems are microphysiological systems that incorporate physiological structure and functionality with human tissues in controlled microenvironments with vasculature‐like perfusion.^272^ These OOC devices introduce another level of complexity to in vitro models, however, this has previously been at the expense of throughput, although there are concerted efforts to increase the throughput of these systems through automation and parallelization of the culture systems.^272^, ^273^ OOC utilizes the power of microfluidics to establish the flow of nutrients, representing the vasculature, while also acting as the culture system (typically microwells or hydrogel) and readout platform.^273^, ^274^ Azizgolshani et al. demonstrated this HT development of an OOC system by creating a system that utilizes a standard 384 well plate and a microfluidic device that allows for the parallel culture of 96 individual OOC systems capable of heterotypic cell culture in one plate. This device featured individually programmable flow control, integrated electrical sensors, and transparent thermoplastic construction that allows for optical access for real‐time in situ readings.^275^ This system was able to perform high‐content analyses of many tissues and could make tissue‐specific readouts. OOC systems are a promising opportunity for tightly controlled HT systems that recapitulate a high degree of the in vivo microenvironment for the modeling of disease, drug screening, predictive toxicity, and personalized medicine.

## 5 CONCLUSION

High throughput systems allow us to empirically test multiple different conditions and factors for their effect on cell behavior. These systems can vary the concentration and combination of soluble and insoluble factors, as well as mechanical parameters such as stiffness and porosity to interrogate their combinatorial effect. With advances in microscale manufacturing and analytical techniques, two‐dimensional high throughput systems have been able to be expanded into the three‐dimensional space allowing for a more in vivo relevant analysis of cellular behavior. We expect with a growing arsenal of microenvironmental modification tools and improvements in culture preparation and analytical methods such as automated handling and imaging, three‐dimensional high throughput systems will become more accessible and poised to support the more advanced analyses of the complex interactions between cells and tissue microenvironmental signals. As these systems become more reproducible and physiologically relevant, we can expect to see faster and cheaper drug development pipelines as well as better yield in directed cell differentiation protocols leading to enhanced personalized medicine and better therapeutic outcomes.

## AUTHOR CONTRIBUTIONS

**Hyeon Ryoo:** Data curation (equal); writing – original draft (equal); writing – review and editing (equal). **Hannah Kimmel:** Data curation (equal); writing – original draft (equal); writing – review and editing (equal). **Evi Rondo:** Data curation (supporting). **Gregory Underhill:** Conceptualization (equal); funding acquisition (equal); project administration (equal); supervision (equal); writing – review and editing (equal).

## CONFLICT OF INTEREST STATEMENT

The authors have no conflicts of interest to disclose.

### PEER REVIEW

The peer review history for this article is available at https://www.webofscience.com/api/gateway/wos/peer-review/10.1002/btm2.10627.

## Data Availability

No primary data was collected as part of this Review Article.
